# A Memoir on the Yellow Fever of the West Indies, *as It Occurred in the Year* 1838, *at St. Pierre, Island of Martinique*

**Published:** 1840-02-01

**Authors:** E. Rufz

**Affiliations:** Adjunct Professor at the School of Medicine of Paris


MEDICAL EXAMINER.

DEVOTED TO MEDICINE, SURGERY, AND THE COLLATERAL SCIENCES.

No. 5.] PHILADELPHIA, SATURDAY, FEBRUARY 1, 1840. [Vol. III.

### A MEMOIR ON THE YELLOW FEVER OF THE WEST INDIES,

*As it occurred in the year* 1838, *at St. Pierre, island of Martinique.* By E. Rufz, D. M. P., Adjunct Professor at the School of Medicine of Paris.

Translated for the Medical Examiner, from the French Manuscript.—[*Continued.*]

CASE VI.

M. de Lucotte, aid-de-camp to the governor, thirty-five years of age, of a robust constitution, and a resident in the colony for six months, led a very irregular life,—walked in the heat of the sun, spent his nights at concerts and suppers, and used Madeira wine very freely. He seemed to brave the disease, trusting to his having lived four or five years in the Morea, where he found the heat more intolerable than in Martinique.

On the evening of the 11th of December I had occasion to be with him: I found him perfectly well. On the 12th, at 7 in the morning, I found him suffering with a severe pain in the head, seated in the middle of the forehead, which he attributed to a sick headach caused by his having been obliged to rise very early, in order to bid adieu to some one, who took his departure that morning. His face was red, his eyes injected, his skin warm, his pulse eighty-four, and full; he was restless, and felt much uneasiness. The development of these symptoms had not been preceded by a chill.

The disease fully declared itself during the day. At 4 o'clock the cephalalgia was insupportable, accompanied with pains in the loins, great heat, and a very full pulse, which was one hundred and sixteen: I took at least sixteen ounces of blood. This bleeding was followed by prostration, which continued for some time. The coagulum was red, not very firm, and without buff; the serum amounted to about a third.

On the night of the 12th he was calm enough; the cephalalgia had diminished, but it resumed its severity on the following day; the face remained flushed, and the eyes injected; there was a slight, but very harrassing cough, with a continual expectoration, and a stopping of the nose; the tongue was natural, slightly whitish; no thirst, a bad taste in the mouth; neither nausea nor vomiting; an almost entire absence of pain in the epigastrium; the abdomen yielding, and free from pain; some stools were obtained by enemata; perspiration very moderate.

During the night of 13th he was very restless, and disturbed by dreams. During the day of the 14th the symptoms were the same, and of the same intensity as on the day before. The restlessness was extreme; the patient changed his bed; the pulse was from eighty-eight to ninety-two, and regular. Towards night the patient began to complain of nausea, and made attempts to vomit.

The night of the 15th he was restless; the symptoms nearly the same; the cephalalgia diminished. There was, at the same time, some cough and expectoration, but there was still extreme agitation; the nausea recurred more frequently. He also vomited some whitish, ropy, and singularly glairy matter. The tongue began to be covered with a yellowish coat; the urine was moderate, clear, and neither scanty nor very abundant; the abdomen yielding; the epigastrium free from pain; the temperature almost natural; the pulse eighty-eight, and regular; the countenance cheerful, and of a lighter colour. Judging by the last two signs only—the countenance and the pulse—one would have thought that the patient was in a state approaching convalescence; but these signs were entirely at variance with the agitation which tormented him.

Though I had watched the patient with some care up to this time, I had discovered no remission, and still less an intermission, in the succession of these phenomena.

After the bleeding the patient was kept on diluents, such as chicken broth, *eau de cerises*, lemonade, sugared water, pure water, &c. Semicupia and injections were used. The night of the 15th he was still very restless; the matter vomited assumed a grayish tint. In the morning thirty leeches were applied to the epigastrium. The leech-bites bled copiously until the next day, when they presented a purple appearance.

On the 16th the nausea and vomiting continued; eleven hours after the application of the leeches there was epistaxis to the amount of half a spoonful; restlessness still considerable; faintings frequent. The patient had an inclination to vomit whenever he moved; pulse seventy-six, full and regular; skin warm and moist; urine less abundant, and more highly coloured, with a slight deposit. The countenance began to assume an expression of suffering.

17*th, the tenth day of the disease.*—Still restless during the night. The matter vomited had commenced to exhibit streaks of a black substance resembling soot; during the whole day there was a constant vomiting of a blackish matter. Towards noon there was, in addition to this, bloody sputa, the source of which was the surface of the tongue, and also the internal surface of the lips. These parts were, in fact, of a deep red colour, and stained the fingers with blood when they were touched; they were not at all swelled, but very sensitive, especially on the contact of acidulous drinks. Soda water produced a burning sensation.

The thirst was more acute than on the preceding day; the abdomen still yielding, and the epigastric region hardly sensible under pressure.

There was constant nausea until death took place, especially when the patient lay on the left side. The paroxysms of vomiting were very frequent, and the matter ejected was stained with black, and with pure blood. The stools were rare, and small, and of a grayish colour. The discharges of urine were few during the day of the 17th, and ceased entirely from the night of the 17th to the 18th.

The cephalalgia had disappeared since the third day; the agitation continued, with rather less pain towards the end. The countenance became more and more expressive of suffering.

There was slight delirium during the night of the 17th. From the 16th the pulse was regular, moderately full, and continued at seventy-six. On the 18th it became more irregular, fell to sixty-eight, and on the approach of death became quite insensible. Syncope became more frequent as the disease advanced.

The skin was soft and moist, and preserved its natural temperature, except at the moment when fainting occurred, at which time it became cold,—only, however, to be immediately restored to its former state.

The continued coldness of the extremities did not commence until the 18th, at 4 o'clock, and from that time continued till death.

Death took place the same day, at 9 o'clock. At the same time that the extremities grew cold, the anxiety of the patient became extreme; the respiration embarrassed, short, and very frequent; the pulse insensible. The nausea and vomiting ceased. His strength was pretty well preserved, for he could raise himself to a sitting posture with considerable quickness. He could not, however, lie on the left side, and complained of intense pain in the præcordial region. At 9 o'clock convulsions came on, and death quickly ensued.

This last evening blisters were applied, and in his last moments sinapisms.

This case presents a very complete group of the symptoms of the fever. It is much to be regretted that an autopsy was not made.

*Anatomical lesions.*—Of nine patients who died, (among those whom I attended, and those on whose cases I was called in consultation,) I have been able to observe the anatomical lesions of three only. Here these cases are reported. For this reason, I shall here confine myself to a few remarks.

The putrefaction of the body after yellow fever is not more rapid than after other acute diseases, other things being equal. The odour is not more decided; and the interment may, without inconvenience, be postponed until twenty-four hours after death.

The yellow colouration took place in these three cases; in two it did not occur until after death. Purple ecchymoses were also formed, especially on the most dependent parts.

In the cavity of the cranium there was fluid black blood in the sinuses; a dark injection of all the vessels of the membranes; little serum under the arachnoid, or in the ventricles. The substance of the brain was injected, and rather firm than softened.

The lungs were filled with dark-coloured blood, especially the posterior half of their inferior lobes. In one case, (that of Sophie,) there was an exudation of blood into the bronchia, and into the tissue of the lung itself. (See case.)

The quantity of serum effused into the pleuræ or pericardium, was never abnormal. M. Catel, physician of the hospital, told me that in one case he found an effusion of blood into the pericardium.

The heart was somewhat flaccid, but not softened; it contained black blood, and sometimes a yellowish clot, small and not adherent. The aorta and the large vessels also contained black blood; their coats were in their natural state, without any red colouring.

The peritonœum contained no effusion; that portion of the membrane which covers the intestines, was sticky and injected.

The stomach contained a greater or less quantity of black matter. This matter dyed the mucous membrane red, but after the latter was washed, it was seen to preserve a rose colour, which was diffused and very delicate, and did not result from a distinct arborescence. Mingled with this colouring, were round spots, formed of black blood, and quite distinct from the rose colour, which might be compared to flea-bites, or to the spots of purpura hæmorrhagica. This mucous membrane was neither thickened nor softened; but it was evidently less tenacious; that is to say, that when attempts were made to detach shreds of it, the shreds broke sooner than they usually do.

The small intestines contained a grayish or whitish matter, particularly the jejunum. Their mucous membrane offered the same appearance as that of the stomach. The purple dotting was more marked, and in the case of one patient, spots were formed as large as the thumb-nail. I suppose that these are the spots which on a superficial examination have been pointed out by authors as traces of gangrene; they are only rare ecchymoses. In some cases the glands of Brunner were here and there of the size of millet seeds; but the glands of Peyer have never presented any modification. I learn from M. Catel, that at the hospital they have the same thing in all the bodies. This fact independently of others, is sufficient to distinguish yellow fever from typhoid.

In one case, the large intestine was the seat of a considerable effusion of blood. In the remainder, its mucous membrane was like that of the stomach and small intestines, bright red, and easily torn without other alterations. The mesenteric glands were a little enlarged, but pretty firm, and almost in their natural state.

As for the liver, I found but twice that yellow colouring, which was the principal lesion in the epidemic of Gibralter. In case eighth, the liver was red, and its vessels contained a great deal of blood, there being no other alteration. I have been informed by M. Catel, who had occasion to make a great many autopsies, that this yellow discolouration of the liver took place in all cases.

The gall-bladder contained little bile; the latter was of a greenish colour. In the case of M. Fraquet, its mucous membrane exhibited a remarkable injection. In the case of Dr. Seisson, we shall see an actual inflammation of this membrane.

The spleen was in one case a little larger than ordinary, and in the other, perfectly natural.

The kidneys were only, like the other organs, filled with fluid bood of a dark colour.

The bladder in one case presented an effusion of blood.

CASE VII.

Sophie, aged twenty-eight years, a native of Champagne, brought as a nurse to Martinique in December last, of a strong constitution, and eminently sanguine temperament. On the 29th of January, she weaned the child which she had been suckling. From the time of her arrival in the colony until this epoch, she had always enjoyed good health.

In spite of my advice to the contrary, Sophie would take salts in order to make her milk pass off; she took about two drachms a day. After the second dose she felt a pain in the head, general uneasiness, and an accession of fever, which she considered as her milk fever. Three days after this, she recommenced taking salts, and she had taken the eighth dose without its having produced any purgative effect, when, on the morning of the 10th February, she was seized with a very severe headach, (without a previous chill) accompanied by redness of the skin, and injection of the conjunctivæ; skin warm; pulse frequent; contusive pain in the limbs; prostration of strength very notable from the commencement; tongue red at the tip, very foul and yellowish at the base; abdomen a little meteorized, and very sensitive to the touch; no evacuations; urine clear and pretty abundant.

(Enemata, pediluvia, lemonade.)

From this moment, the symptoms were more and more developed.

The cephalalgia, after having lasted with great severity for forty-eight hours, disappeared, and was replaced by great dulness.

On the 11th, the second night of her disease, there was delirium, her sleep was much disturbed. The drowsiness was constant; the prostration of strength very notable.

The second day she vomited some whitish matter.

On the third and fourth (12th and 13th of February) the vomiting recurred two or three times a day, the matter ejected being always of a whitish colour; it did not exist on the subsequent days.

Her thirst, which had been almost inappreciable, until the third day, became intense and continued until death. At the same time, the tongue grew redder at the tip, and appeared to have a tendency to become dry.

The abdomen continued all the time meteorized and very sensitive.

Up to the fourth day, only one or two stools a day had been obtained by injections. On the fourth day (the 13th February) she had ten stools of a whitish colour, resembling rice custard. The following day these evacuations became very frequent and almost involuntary; the stools assumed a red tint.

On this day her urine was suppressed, and did not re-appear.

Her pulse, which, during the first two days was 100, full, soft and regular, fell on the third day to 84; it kept at this, maintaining its regularity during two days, and became insensible some hours before death.

The heat of skin which was at first considerable, became very moderate during the third and fourth days. The extremities became cold seven hours before death. The sweats which took place the first day offered nothing remarkable.

We did not observe any appreciable remission in the progress of the disease.

The drowsiness and prostration of strength which were manifested at the commencement of the attack, went on increasing continually. There was, however, great restlessness; the patient rolled from one side of the bed to the other, without being able to remain in any one place.

The face which was red and flushed the first two days, grew a little pale, then became purple, and from the third day assumed a contracted expression.

The prostration of strength had prevented my having recourse to general bleeding. The first day I applied thirty leeches behind the ears; and on the second day the same number to the epigastrium. The third day, blisters to the legs, and three spoonfuls of castor oil. It was after the exhibition of the oil, that the diarrhœa made its appearance.

Death took place at the close of the fifth day.

*Examination three hours after death.*—The body was still warm, and without rigidity; fulness considerable; all the tissues presented a jaundiced appearance. From the scalp and dura mater oozed dark blood. The pia mater was every where, at the base as well as on the convex surface of the brain, injected with blood of a dark rust colour. The substance of the brain was firm and injected in the same manner. The ventricles contained two spoonfuls of serum.

The two lungs adhered to the pleuræ by some old adhesions. Both were crepitant, but their posterior portions presented black spots, which resulted from an infiltration of blood, which formed kernels such as occur in apoplexy of the lungs. The bronchiæ were filled with extravasated black blood, and their mucous membrane was also of a dark colour, in consequence of an infiltration of the same kind.

If the patient had lived a little longer, it is probable that she would have had a violent hæmoptysis.

The tissue of the heart was firm; the cavities contained dark blood, without a clot; no particular modification. The aorta and venæ cavæ also contained black blood, which was without a clot: their tissue was not coloured red.

The pleura and pericardium contained hardly a spoonful of serum.

The peritoneum contained no serum; the portion covering the intestines was very much injected.

The stomach was somewhat dilated, and contained two or three spoonfuls of a liquid bearing a close resemblance to old wine. This liquid gave to the mucous membrane a shadowing which was removed by washing: but the latter, notwithstanding, preserved a red colouring quite distinct from that which resulted from the imbibition: this coloration was general. The mucous membrane was not, however, softened; it was more frail, and yielded shreds which broke easily.

In the small intestines, there was no effusion of blood; but their mucous membrane was coated with a whitish mucus, and presented the same red tint as that of the stomach; it was frail, without true *ramollissement.*—The glands of Peyer were slightly injected, but were neither enlarged nor ulcerated. The glands of Brunner were not visible.

The large intestine offered the same appearance as the small.

The mesenteric glands were in their natural state. It was the same case with the spleen, which was consistent, and by no means of a large size.

The liver was of a deep reddish-purple colour, and distilled a great deal of blood; its tissue was neither softened nor otherwise modified. The gall bladder was unchanged, and contained a little bile deeply tinged with green.

The kidneys were filled with blood. The bladder was contracted, but unchanged. The uterus was very much injected; but there was no effusion of blood into it.

CASE VIII.

I was called upon, on the 30th of January, by Dr. Lagrange, to make, with him, an autopsy of an American captain, who had died of yellow fever.

This man, who came last from Demarara, fell sick during the passage, four or five days before his arrival at Martinique. He was brought ashore by his own directions, and two days after, he died. He had had a high fever, pain in the head, icterus, and vomiting of black matter, but no diarrhœa.

The autopsy was made two days after death.

The lungs contained air, except at the posterior part, which presented patches infiltrated with dark blood. The bronchi were of a purple colour, but there was no blood effused into their cavity.

The heart was in its natural condition; its tissue was firm, and its cavities filled with dark blood. There was neither effusion into the pleuræ or pericardium.

The stomach was distended by about twelve or thirteen ounces of a black fluid, which was recognised to be blood, almost pure. Its mucous membrane was generally of a bright red, without any particular injection. It was fragile throughout, but not softened,—that is to say, that the shreds which we attempted to detach were of some length, but broke very readily.

The mucous membrane of the large and small intestines was in the same condition. It would have been pronounced to be an erythema of the whole membrane of the intestines. The glands of Peyer and of Brunner were not apparent. There was no effusion of blood into their cavity.

The liver was yellowish, like Spanish tobacco, and of the ordinary size and consistence. The gall-bladder was contracted in itself, and contained a little bile, which was thick, and of a greenish colour; its mucous membrane was natural. The spleen was a fourth larger than ordinary, but not softened.

The kidneys and bladder offered nothing remarkable. The brain was not examined.

Such were the anatomical lesions. It is seen that the modification of the liver holds the first rank: those of the stomach and intestines the next. All the organs were the seat of sanguineous congestion; but hæmorrhages were not as frequent as they have usually been said to be. The appearance of the blood evinced a great alteration. We repeat here, what we have already said, viz., that after death, as well as during life, we find nothing which should confound this disease with the typhoid fever of Europe.

(*To be continued.*)

